# Developing and validating a hand hygiene skills intervention for Primary Health Care nurses

**DOI:** 10.4102/safp.v68i1.6364

**Published:** 2026-10-06

**Authors:** Nokwazi I. Dlamini, Thembelihle S.P. Ngxongo, Nellie Naranjee, Jeffrey M. Hadebe

**Affiliations:** 1 Department of Nursing, Faculty of Medicine and Health Sciences, Walter Sisulu University, Mthatha, South Africa; 2 Department of Nursing, Faculty of Health Sciences, Durban University of Technology, Durban, South Africa

**Keywords:** hand hygiene, infection prevention and control, Primary Health Care, nursing education, skills-based training, Delphi technique, South Africa

## Abstract

**Background:**

Hand hygiene is a cornerstone of infection prevention and control in Primary Health Care (PHC) settings. Despite established guidelines, compliance among PHC nurses in South Africa remains suboptimal. Practical, context-specific educational strategies are needed to strengthen routine practice. This study aimed to develop and validate a skills-based hand hygiene educational intervention suitable for PHC nurses.

**Methods:**

A development-and-validation study design was used. A hand hygiene skills-based educational intervention was developed using evidence, infection prevention and control guidelines and findings from prior exploratory work. The intervention was validated using a three-round Delphi technique with 12 experts in infection prevention and control, healthcare education, public health and quality improvement. Consensus was used to assess the relevance, feasibility and educational suitability.

**Results:**

Consensus was achieved across all Delphi rounds on the validity and appropriateness of the intervention for PHC settings. All five components of the skills-based intervention achieved mean importance and effectiveness ratings of ≥ 4.0 on a five-point Likert scale. Six themes emerged: interactive learning; clinical contextualisation; immediate feedback; regular reinforcement; institutional culture and leadership support; and sustainability.

**Conclusion:**

The study produced an expert-validated hand hygiene intervention for PHC nurses. The intervention provides a practical educational framework that for integration into existing infection prevention and control programmes in PHC clinics.

**Contribution:**

Further research is required to evaluate its impact on hand hygiene practice and patient outcomes.

## Introduction

Healthcare-associated infections (HAIs) remain a significant and largely preventable threat to patient safety within healthcare systems. In South Africa, this burden is particularly evident in Primary Health Care (PHC) settings, which serve as the first point of contact for most patients and operate under conditions of high patient volumes, staff shortages and limited resources.^1^ Nurses form the backbone of PHC service delivery and are central to infection prevention and control (IPC) efforts. Ensuring safe, consistent infection-prevention practices in this context is therefore essential for improving the quality of care.

Hand hygiene is universally recognised as the most effective and cost-efficient measure for preventing the transmission of infection in healthcare settings. International and national guidelines consistently identify hand hygiene as foundational to IPC, with a benchmark compliance target of at least 80%.^2^ Despite this, evidence from low- and middle-income countries, including South Africa, indicates that hand hygiene compliance among healthcare workers remains suboptimal, particularly in PHC settings.^3^ Reported compliance rates in some South African clinics fall well below recommended standards, placing patients at risk of avoidable infection and associated complications.^3^

Poor hand hygiene compliance among nurses has been attributed to a range of interacting factors. These include high workload and time constraints, inconsistent access to hand hygiene resources, limited opportunities for refresher training, habitual practice patterns and workplace cultures in which hand hygiene is assumed rather than actively reinforced.^4,5^ Importantly, awareness of hand hygiene guidelines does not necessarily translate into sustained observable practice. This persistent gap between knowledge and behaviour suggests that traditional training approaches, often delivered as once-off, lecture-based sessions, are insufficient to support meaningful and lasting change in everyday clinical practice.^4^

Educational interventions are a key modifiable component of strategies to strengthen hand hygiene practices. Evidence indicates that educational approaches are more effective when they are interactive, contextually relevant and reinforced over time.^4,6^ Interventions that allow healthcare workers to practise skills, receive immediate feedback and engage with peers are more likely to influence behavioural change than passive information-based teaching alone.^6^ Within PHC settings, where nurses spend most of their time providing direct patient care, educational strategies that emphasise practical, skills-based learning are particularly appropriate.

Skills-based educational interventions, such as skills fairs, have gained increasing attention in healthcare education for their ability to deliver focused, experiential learning in a time-efficient manner.^7,8^ Skills fairs typically consist of short, rotating stations that address specific competencies, enabling participants to engage in hands-on practice, observe peer performance and receive facilitated feedback. This format is well-suited to PHC settings, where releasing staff for prolonged training can disrupt service delivery and where educational activities need to be flexible and adaptable.^7,8^

Validation is an important but often neglected step in the development of educational interventions. Expert validation helps ensure that an intervention’s content is relevant, comprehensive and appropriate for its intended audience, and that it aligns with current evidence and practice requirements.^9^ The Delphi technique is a well-established method for achieving structured expert consensus and has been widely applied in the development of educational programmes, clinical guidelines and healthcare interventions.^10^ By incorporating multidisciplinary expert perspectives, validated interventions are more likely to be credible, acceptable and feasible for implementation in real-world clinical settings.^9,10^

Despite increasing interest in skills-based learning, few hand hygiene interventions have been specifically developed and validated for PHC nurses in South Africa. Many programmes are adapted from hospital settings and may not adequately address the realities of PHC practice. This highlights the need for interventions tailored to the PHC context.^11^

This study addresses the need for a contextually appropriate and rigorously developed educational strategy to strengthen hand hygiene practices among PHC nurses. Given the importance of establishing content validity before implementation, expert validation was undertaken as a critical step in intervention development. Rather than evaluating intervention effectiveness, which will be assessed in a subsequent implementation phase, this study focused on the structured development and expert validation of a skills-based hand hygiene educational intervention designed for routine use in South African PHC settings.

### Aim

The aim of this study was to develop and validate a hand hygiene skills intervention suitable for PHC nurses, using expert consensus to assess its content, structure, feasibility and educational suitability within the South African PHC context.

## Research methods and design

### Study setting

The intervention was developed for use in public PHC clinics in KwaZulu-Natal, South Africa. The intended implementation context includes urban, peri-urban and rural PHC facilities providing comprehensive healthcare services under varying resource constraints. The intervention was designed to accommodate the operational realities commonly encountered in these settings.

### Study design

A development and validation study design was employed to develop and validate a hand hygiene skills-based educational intervention for PHC nurses. The study focused on intervention development and expert validation as a preliminary phase in intervention design. Evaluation of implementation outcomes and intervention effectiveness was beyond the scope of the current study and will be explored in future research.

### Development of the hand hygiene skills intervention

The intervention was developed through a structured process informed by the literature on hand hygiene education, IPC guidelines, principles of adult learning and findings from an earlier exploratory mixed-methods phase of the broader doctoral study (unpublished doctoral research), which was used to guide content selection and contextual adaptation.^11^

The intervention was designed as a skills-based educational programme delivered in a skills fair format, selected for its suitability within high-workload PHC environments. The programme comprised five interactive stations addressing key components of hand hygiene practice: (1) handwashing technique, (2) the chain of infection and the role of hand hygiene, (3) the World Health Organization’s (WHO) five moments of hand hygiene, (4) types of hand hygiene and clinical decision-making and (5) peer learning supported by educational resources. Each station was designed with clearly defined learning objectives and included demonstrations, practical activities, opportunities for skills practice and immediate feedback. Educational resources and structured facilitation guides were incorporated to support participant engagement and learning. The intervention was structured to promote experiential learning, peer engagement and the application of knowledge within routine PHC practice.

### Validation method

The developed intervention was subjected to expert validation using a three-round Delphi technique, a widely accepted method for obtaining structured consensus among experts on complex healthcare and educational interventions.^9,10,12^

The Delphi process was selected to assess the relevance, clarity, feasibility and educational appropriateness of the intervention components for use in South African PHC settings.^10^

The Delphi technique is a well-established method for achieving structured expert consensus and has been widely applied in the development of educational programmes, clinical guidelines and healthcare interventions.^10^ The technique facilitates anonymous, iterative feedback and controlled consensus-building, making it particularly useful when expert judgement is required to inform healthcare decision-making and intervention development.^12^ By incorporating multidisciplinary expert perspectives, validated interventions are more likely to be credible, acceptable and feasible for implementation in real-world clinical settings.^9,10^

### Expert panel selection

A purposive sampling strategy was used to recruit a multidisciplinary panel of experts with relevant experience in IPC, healthcare education, public health and quality improvement. Inclusion criteria were professional expertise and experience in developing, implementing or evaluating IPC or educational initiatives in healthcare settings. Experts were excluded if they had less than 5 years of relevant professional experience, lacked current involvement in IPC, healthcare education, public health or quality improvement activities, or were unable to participate in all Delphi rounds.

The final panel comprised 12 experts: five infection control specialists, three healthcare educators, two public health officials and two quality improvement practitioners. This composition was intended to ensure balanced input across clinical, educational and system-level perspectives. Experts represented a range of healthcare settings, including urban, peri-urban and rural service environments.

### Delphi procedure

The Delphi process was conducted using a hybrid approach that combined electronic and offline data collection to optimise participation and accommodate participant preferences and logistical constraints, while maintaining standardised procedures across all rounds.

#### Round 1

Round 1 consisted of an open-ended questionnaire in which panellists were asked to evaluate the relevance, completeness, feasibility and educational suitability of the intervention. They were also invited to identify strengths, weaknesses, missing content areas and implementation considerations relevant to PHC settings. Responses were analysed thematically and used to refine the intervention and develop structured rating items for round 2.

#### Round 2

Round 2 involved a structured questionnaire in which panellists rated the importance and anticipated effectiveness of each intervention component using a five-point Likert scale ranging from ‘not important’ to ‘extremely important’. Consensus was defined *a priori* as a mean score of ≥ 4.0.

#### Round 3

Round 3 provided panellists with anonymised feedback from round 2 and invited recommendations for enhancing the intervention. Final adjustments were made based on converging feedback.

### Data analysis

Qualitative data from Delphi procedure rounds 1 and 3 were analysed using thematic analysis. Quantitative data from round 2 were analysed descriptively to determine levels of consensus. All analyses focused on assessing the validity, educational suitability and feasibility of the intervention, rather than outcome effectiveness.

### Ethical considerations

Ethical approval was obtained from the Institutional Research Ethics Committee (IREC 157/24). Permission to access PHC facilities was granted by the KwaZulu-Natal Department of Health and relevant district authorities. All expert panellists provided informed consent before participation. Anonymity and confidentiality were maintained throughout the Delphi process.

## Results

### Expert panel characteristics

A total of *N* = 12 experts participated in the Delphi validation process. The panel comprised (*n* = 5) IPC specialists, (*n* = 3) three healthcare educators, (*n* = 2) public health officials and (*n* = 2) quality improvement practitioners. All panellists had professional experience relevant to hand hygiene initiatives or educational programme development within healthcare settings. Consensus was achieved across the three Delphi rounds on the overall validity, relevance and educational suitability of the hand hygiene skills intervention for PHC nurses. All five components of the intervention met the predefined consensus threshold of a mean rating of ≥ 4.0 on a five-point Likert scale.

### Round 1: Qualitative feedback on the proposed intervention

#### Qualitative themes emerging from expert feedback

Round 1 generated qualitative feedback regarding the relevance, completeness, feasibility and educational suitability of the proposed intervention. Thematic analysis of panellist responses yielded six overarching themes that reflected expert perspectives on the intervention’s strengths and suitability.

#### Interactive learning is essential

Experts consistently emphasised the importance of hands-on, interactive learning approaches in strengthening hand hygiene practices. The skills-based station format was viewed as particularly appropriate for reinforcing correct technique and actively engaging PHC nurses in the learning process.

#### Clinical contextualisation

Panellists highlighted the value of aligning training content with everyday PHC clinical scenarios. Context-specific examples and role-play activities were considered critical for improving the relevance and transferability of learning to routine practice.

#### Immediate and constructive feedback

The provision of real-time feedback during skills demonstrations, particularly during handwashing technique sessions, was identified as a key strength of the intervention. Experts noted that immediate feedback supports skill correction and reinforces correct practice.

#### Regular reinforcement

The proposed biennial delivery of the intervention was viewed as a practical approach to maintaining hand hygiene awareness while minimising disruption to service delivery. Several panellists highlighted the importance of complementary reinforcement activities between training sessions.

#### Institutional culture and leadership support

Experts emphasised that the intervention’s long-term success would depend on visible leadership support and integration within existing IPC and quality improvement activities. Alignment with facility-level governance processes was considered important for sustaining behavioural change.

#### Sustainability and feasibility

Panellists recognised the intervention as feasible within PHC settings, provided that appropriate planning and facilitation were in place. Suggestions included structured facilitation guides and access to educational resources to support sustainability.

### Round 2: Consensus ratings of intervention components

Consensus was achieved on the overall validity, relevance and educational suitability of the hand hygiene skills intervention for PHC nurses. All five intervention components exceeded the predefined consensus threshold of a mean rating ≥ 4.0 on a five-point Likert scale. Table 1 summarises the mean importance and effectiveness ratings for each intervention component

**TABLE 1:** The mean importance and effectiveness ratings for each intervention component.

Intervention component	Mean importance rating (1–5)	Mean effectiveness rating (1–5)	Consensus achieved
Handwashing technique	4.9	4.7	Yes
Chain of infection and role of hand hygiene	4.7	4.5	Yes
Five moments of hand hygiene	4.9	4.8	Yes
Types of hand hygiene	4.6	4.4	Yes
Educational materials and peer learning	4.5	4.1	Yes

The highest-rated intervention components were handwashing technique and the WHO five moments of hand hygiene, both achieving mean importance ratings of 4.9. Educational materials and peer learning received the lowest ratings; however, these remained above the consensus threshold, with mean importance and effectiveness ratings of 4.5 and 4.1, respectively. These findings indicate strong overall agreement regarding the relevance and educational value of all intervention components.

Panellists rated all components as either ‘very important’ or ‘extremely important’ for inclusion in a PHC-focused educational intervention.

### Round 3: Intervention refinement and validation

Round 3 focused on achieving a final consensus and refining intervention content based on structured feedback from previous rounds. Several modifications were incorporated into the final intervention, including the development of a structured facilitation guide for peer-learning activities, clearer alignment with facility IPC audit processes, incorporation of post-training mentorship support and inclusion of supplementary educational resources for ongoing access.

## Discussion

This study describes the development and expert validation of a skills-based educational intervention to strengthen hand hygiene practices among PHC nurses. Using a structured Delphi process, consensus was achieved on the relevance, educational suitability and feasibility of the intervention components for use in South African PHC clinics. These findings indicate that the intervention is contextually appropriate and aligns with current expectations for infection prevention education in PHC settings.

The strong consensus achieved across all five intervention components underscores the importance of practical, skills-focused approaches to hand hygiene education. Suggestions included structured facilitation guides and access to supporting educational resources, including both digital and printed materials, to enhance sustainability and accessibility across diverse PHC settings. Experts consistently endorsed interactive learning, immediate feedback and clinical contextualisation as essential elements for improving hand hygiene practices. This aligns with existing evidence demonstrating that multimodal, experiential educational approaches are more effective than didactic methods in influencing healthcare worker behaviour and improving hand hygiene compliance.^6,13,14^ For PHC nurses, whose work is characterised by high patient turnover and competing service demands, short, focused training activities that mirror routine practice are particularly relevant.

The highest-rated intervention components were handwashing technique and the WHO five moments of hand hygiene, both receiving mean importance ratings of 4.9. These findings emphasise the importance that experts placed on practical competency development and adherence to evidence-based hand hygiene guidance. Although educational materials and peer learning received comparatively lower ratings, consensus remained high, indicating that experts considered these components valuable for supporting ongoing learning and reinforcement.

Experts highlighted the importance of tailoring hand hygiene education to everyday PHC practice. Role-play scenarios and demonstrations based on real clinical situations were considered more likely to support the application of learning in practice. This is consistent with evidence showing that contextually relevant educational interventions are more effective in promoting sustained behaviour change.^4,5^ These findings support the need for PHC-specific hand hygiene education rather than relying exclusively on programmes developed for hospital settings.

Immediate feedback was identified as one of the key strengths of the intervention, particularly during the handwashing technique component. This observation is consistent with educational literature demonstrating that feedback provided during skill performance supports learning, skill acquisition and retention.^6^ Immediate feedback may therefore strengthen the educational value of hand hygiene training by facilitating timely correction of technique and reinforcing correct practice.

Experts emphasised the importance of regular reinforcement. Although biennial delivery of the intervention was considered practical within PHC operational constraints, panellists highlighted the need for complementary reinforcement activities between training sessions. Evidence from implementation science suggests that sustained improvements in clinical practice are more likely when educational interventions are supported by structured organisational change processes and ongoing reinforcement mechanisms.^15^ Future implementation of the intervention should include structured evaluation of participant reactions, learning, behavioural change and organisational outcomes to determine its effectiveness in practice.^16^

This finding aligns with the WHO multimodal hand hygiene improvement framework, which identifies education as one component of a broader strategy that includes audit, feedback, reminders and institutional safety culture.^17^

Institutional culture and leadership support were consistently identified as determinants of the intervention’s long-term success. Educational interventions alone are unlikely to produce sustained behavioural change in the absence of visible organisational commitment to infection prevention. Sustained practice change requires implementation strategies that include leadership engagement, reinforcement and integration into routine organisational processes.^15^ Integration of hand hygiene education within routine IPC governance, leadership endorsement and alignment with quality improvement processes has been shown to be critical for maintaining compliance over time.^13,17^

The inclusion of both digital and printed educational resources was considered important to support continued learning and improve accessibility across different PHC settings. Flexible access to educational materials may help reinforce key messages and support the long-term sustainability of the intervention.

### Implications for Primary Health Care practice

This expert-validated intervention offers PHC managers, family physicians and nurse leaders a structured, feasible educational model that can be incorporated into existing IPC programmes. The skills-based format allows for flexible implementation without prolonged disruption to service delivery, making it suitable for busy clinic settings. The intervention also supports standardisation of hand hygiene education across facilities while allowing contextual adaptation based on local needs, audit findings and resource availability. Future implementation of the intervention should include structured evaluation of participant reactions, learning, behavioural change and organisational outcomes to determine its effectiveness in practice.^16^

### Strengths and limitations

A key strength of this study was the inclusion of a multidisciplinary expert panel representing IPC, healthcare education, public health and quality improvement perspectives. This diversity enhanced the content validity, contextual relevance and practical applicability of the intervention. The use of a structured three-round Delphi technique facilitated systematic consensus building and iterative refinement of intervention content, contributing to the methodological rigour of the validation process. Furthermore, the intervention was informed by findings from an earlier exploratory mixed-methods phase, ensuring that its content was grounded in the realities of PHC nursing practice.

Despite these strengths, several limitations should be acknowledged. Firstly, the Delphi panel was relatively small and purposively selected, which may limit the generalisability of findings beyond similar contexts. Secondly, although experts represented a range of relevant disciplines, their views may not fully reflect the perspectives of all PHC nurses who will ultimately use the intervention. Thirdly, this study focused exclusively on intervention development and expert validation and did not evaluate implementation, effectiveness, behavioural outcomes or patient-related outcomes. Consequently, the intervention’s ability to improve hand hygiene compliance in practice remains unknown. Future research should therefore assess the feasibility, acceptability and effectiveness of the intervention in PHC settings and determine its impact on hand hygiene practices and patient safety outcomes.

## Conclusion

This study developed and validated a skills-based hand hygiene educational intervention tailored to the realities of South African PHC clinics. Expert consensus supports the intervention’s relevance, feasibility and educational appropriateness for PHC nurses. While not intended as a standalone solution, the intervention provides a structured, validated framework that can be integrated into broader IPC strategies to support safer care in PHC settings.
